# Evaluation of the release of nickel and titanium under orthodontic treatment

**DOI:** 10.1038/s41598-020-79221-1

**Published:** 2020-12-17

**Authors:** Rafael Velasco-Ibáñez, Edith Lara-Carrillo, Raúl Alberto Morales-Luckie, Elizabeth Teresita Romero-Guzmán, Víctor Hugo Toral-Rizo, Marius Ramírez-Cardona, Verónica García-Hernández, Carlo Eduardo Medina-Solís

**Affiliations:** 1 Facultad de Odontología, Universidad Autónoma del Estado de México, Av. Paseo Tollocan esq. Jesús Carranza. Colonia Universidad, C. P. 50130 Toluca de Lerdo, Mexico; 2 Centro de Investigación en Química Sustentable, CIQS, UAEM-UNAM, Universidad Autónoma del Estado de México, Carretera Km. 14.5, Unidad San Cayetano, Toluca–Atlacomulco, C. P. 50200 Toluca de Lerdo, México; 3 Departamento de Química, Gerencia de Ciencias Básicas, Instituto Nacional de Investigaciones Nucleares, Carretera México-Toluca S/N, La Marquesa, C. P. 52750 Ocoyoacac, Mexico; 4 Área Académica de Odontología, Instituto de Ciencias de la Salud. Universidad Autónoma del Estado de Hidalgo, San Agustín Tlaxiaca, Hidalgo Mexico

**Keywords:** Fixed appliances, Adverse effects, Predictive markers

## Abstract

The metal alloys used in dentistry are made mainly of nickel (Ni), titanium (Ti), and other elements such as molybdenum (Mo), zirconium (Zr), iron (Fe), tin (Sn), chrome (Cr), carbon (C), copper (Cu) and niobium (Nb) which can release metal ions in unstable environments. The aim of this work was determine the salivary pH before and during orthodontic treatment; evaluate the release of metal ions, mainly Ni and Ti, in urine and saliva using Inductively Coupled Plasma Optical Emission Spectroscopy (ICP-OES); and evaluate the corrosion using Scanning Electronic Microscopy (SEM). In this study, we selected 35 individuals under orthodontic treatment, from whom saliva and urine samples were collected in 3 stages: (a) basal, (b) at 3 and (c) 6 months after the placement of the fixed appliances. SEM analyzed the Ni–Ti (0.016″) and stainless steel (SS) (0.016 × 0.022″) archs after 1 month of being in contact with the oral cavity. Statistical analysis was performed with Stata using the ANOVA model of repeated measures with a p < 0.05. A statistically significant difference in the concentration of Ni in saliva were found between 3 and 6 months of intervention and Ti in urine was found 3 and 6 months.

## Introduction

Orthodontics is responsible for the diagnosis, planning, and evaluation of the therapeutic result of dental malocclusions through the use of attachments such as arches, brackets and bands. These exert biomechanical forces that perform tooth movements and improve the function and aesthetics of the individual^1–4^.

The metallic alloys used in dentistry contain nickel (Ni), cobalt (Co), chromium (Cr), titanium (Ti), iron (Fe), and copper (Cu). There are four major orthodontic alloys in current widespread use for orthodontic wires, brackets, and other orthodontic appliances such as stainless steel (Fe-Cr-Ni), cobalt-chromium (Co-Cr-Fe-Ni), nickel–titanium (Ni–Ti), and beta-titanium (Ti-Mo-Sn-Zr) and consist of approximately 15–54% of Ni, 20–30% of Cr, and 40–60% of Co^5–7^. The most important selection factor regarding orthodontic attachments is the biocompatibility of materials. The AISI type 316L austenitic stainless steel is the most commonly used orthodontic bracket and arch wire material. It is composed (wt%) of 0.08 carbon, 2.00 manganese, 0.045 phosphorus, 0.03 sulfur, 0.75 silicon, 16 to 18 chromium, 10 to 14 nickel, and 2 to 3 molybdenum, with the remainder being iron^8^. Biocompatibility is the ability of a biomaterial to perform a desired function with respect to a person, without causing undesirable local or systemic effects^9^. The most important characteristic of biomaterials in orthodontics is the resistance to corrosion^10^. Nickel is a nutritionally essential trace element for some animals, microorganisms, and plants. Although its function and vitality of humans has not been confirmed, it is known that people eat about 100 to 300 µg per day^11^. However, a concentration of 0.78 ng mL^−1^ could generate cellular damage in the oral mucosa^12,13^. Some adverse effects of Ni are the induction of local inflammation, type IV hypersensitivity, allergic contact stomatitis, allergic reactions, and cancerous/mutagenic actions^14,15^. For its part, titanium is widely used in dental materials due to its excellent biocompatibility and resistance to corrosion^16^.

The Ni–Ti wire arches are commonly used in orthodontics because of their elastic capacity and resistance to the corrosion mainly in the form of arch wires for the initial levelling phase of treatment. The alloy used for these wires consists of approximately 55% Ni and 45% Ti and may contain small amounts of Cu or other elements^8^. The several modifications increased desired properties such as low stiffness, shape memory, and superelasticity. In addition, the high springback property of nitinol permits the delivery of low forces even at large deflection, which is particularly useful in cases with severe crowding. They are useful because they allow the application of constant forces during a long period of activation^17,18^. However, one of the main problems related to the use of Ni–Ti alloy as a biomaterial is the release of carcinogenic Ni and Ti ions into the human body^19^. In order to evaluate the degree of bioaccumulation of these metal ions in the organism, both the basal exposure and its evolution in the time of treatment among biological samples are used. Among the biomarker noninvasive exposure, such as biological matrix include the following: hair, saliva, urine, and nails. These biological samples are most widely used because of their easy disponibility and accuracy assessment^20–22^. Metallic corrosion is an electrochemical process that generates loss of material due to a reaction of the metal in contact with an aqueous solution. The chemical composition of the aqueous medium determines the extent of the corrosion process^23^. In the oral cavity, there are many factors that increase the biodegradation of orthodontic appliances by different conductors of corrosion^24^. Saliva acts as a lubricant and aids chewing by facilitating food transport^25^. In addition, it functions as an electrolyte for ion conduction. Fluctuating pH, temperature, enzymatic, and microbial activity introduced into the oral cavity through food and beverages generate mechanical degradation and chemical degradation. The devices used in orthodontic metal (e.g., wires, tubes, bands) remain in the mouth for an average of 2 years in this potentially corrosive environment^26^.

Therefore, the objective of this research was to evaluate the release of metal ions in the mouth by the use of fixed appliance orthodontic by ICP-OES in urine and saliva, and to determine the salivary pH before and during orthodontic treatment. And to evaluate the morphological changes of orthodontic archs with SEM.

## Materials and methods

The present study is a longitudinal comparative study that was performed in the specialty clinic in orthodontics of the Autonomous University of the State of Mexico.

### Participants

35 individuals (21 female and 14 male) were selected. The age of them range from 11 to 17 years old, all were under orthodontic treatment. For all of them, informed written consent was obtained from a parent or legal guardian prior to the experiment, and they were informed about the research objectives to continue investigation. Participants had to meet the following criteria: patients without any metallic restoration in the mouth, patients with permanent dentition and without systemic diseases.

### Characterization of the population

All participants completed a questionnaire aimed at the objectives obtaining for the people who explain the sociodemographic characteristics and health habits of the patients.

### Sample collection and processing

High density polyethylene (HDPE) flasks (mod. AQ 001; ítem 912, Kartell_SpA_ divisione labware) were washed with soap and Alkali Extran (Merck) and allowed to soak in 5% hyperpure nitric acid (v/v) for 24 h to avoid background contamination with heavy metals. Finally, they were washed with double- distilled water and dried at environmental temperature ready to collect the samples.

For the sampling of saliva, the following indications were given to each patient: Do not ingest food or beverages before sampling; rinse mouth with potable water and wait 3 min. 5 mL of stimulated saliva were collected for 5 min in the polyethylene bottles directly when the patients arrived at the orthodontic treatment and stored at – 4 °C until they were processed. This procedure was carried out in the 3 phases: (a) the first measurement before the treatment; (b) the second measurement 3 months after placement of fixed appliances (e.g., wires, tubes, bands), and (c) the third measurement at 6 months after the beginning of the treatment. 50 mL of the first morning urine from each patient were placed in polyethylene containers trying to avoid, contaminating the collection vessel. This procedure was performed in all phases. Saliva and urine samples were placed in 5% nitric acid (v/v) for the samples preservation, and they were kept refrigerated at 4 °C for, their processing, and analysis with ICP-OES.

Ni–Ti heat activated wires (3 M™ Unitek™ mark) with 0.016″ gauge and 0.016 × 0.022″ stainless steel wires (3 M™ Unitek™ mark) were collected after 4 weeks of intraoral use during the orthodontic treatment. They were compared with a new reference an arch without contact with the oral environment to correlate the archs exposure without and with exposure to the oral environment. Ni–Ti wires were used in patients who were in the leveling stage within orthodontic treatment due to the flexibility it provides and the irregularity of the patient teeth. Subsequently, the arches used were of stainless steel with different calibers, concluding the treatment with the 0.017 × 0.025″ caliber. The arches were cut with pliers for ligature cutting, and the length of each segment was of 3 mm for each arch. Two different areas of the Ni–Ti arch (TP Orthodontic mark) and stainless steel arch (TP Orthodontic mark) were taken in a distal and in a middle portion to represent a differential area for each arch. Then the archs were analyzed by SEM. In saliva samples the pH was determined using a potentiometer SperScientific Model 840088.

### Digestion of samples

4 mL of urine and saliva were transfered into the digestion vessel for each one. 5 mL of ultrapure grade HNO_3_ and 2 mL of H_2_O_2_ were added. The mixture was gently swirl and waited approximately 15 min before closing the Teflon vessel. The sampled was digested until 200 °C during 30 min in a MARSX press microwave (CEM). With this digest we break the bond between organic matter (such as proteins) and nickel. Then the samples were diluted with 50 mL of double demineralized water (Millipore Simplicity).

Multielemental analysis by ICP-OES: The analysis was performed with an ICP-OES OPTIMA 8300, Perkin Elmer. For the determination of Ni and Ti a Certified Reference Materials (CRM) of Ultra Scientific brand element were used. The calibration curve included the following concentrations: 0.01, 0.025, 0.05, 0.1, 0.5 and 1 mg L^−1^ in a matrix ultrapure grade 2% nitric acid.

Each saliva and urine sample was analyzed in the same conditions of calibration solutions in triplicate, and the average was used as the result. As well, the blank measurements values (MilliQ water) were substracted from the results obtained for the samples.

The detection limit for Ni was of 0.1–1 ng mL^−1^ and for the Ti was ≤ 0.1 ng mL^−1^.Similar procedure performed by Petoumenou et al*.*^27^.

### Analysis of SEM and EDS

The 0.016″ Ni–Ti and 0.016 × 0.022″ stainless steel arches were placed on aluminum sample holder and using a conductive carbon tape. The arches were grouped, including control samples. Three arcs were included in each group, and these arcs were analyzed by SEM. The elemental chemical composition of the samples was determined using an energy dispersive X-ray spectrometer (EDS) with a JSM-6510 Jeol-LV-20 kV accelerating secondary electrons and to examine them.

### Analysis of the information

Statistical analysis was performed using software Stata 14. The analysis comprises two steps (i.e., statistical and descriptive) to better understand the behavior of the variables studied, and repeated measures ANalysis Of VAriance (ANOVA) calculations were used to establish statistically significant differences by group of intervention between the 3 stages of the study: basal, 3 and 6 months after the placement of fixed orthodontic appliances. Prior to the bivariate analysis, the assumptions for the ANOVA test were tested. To detect outliers, the test was performed^28,29^ this test can be used to detect outliers in a data set. In Shapiro–Wilk W test for normal data we observed that for titanium in saliva the data were distributed in a non-normal way, so that we implements the Skillings-Mack (SM) test, which is a generalization of the Friedman test. Due to the difficulty of complying with the assumption of spherity (the variances of the differences between all combinations of related groups must be equal), it was decided to use the correction of p-value in ANOVA. Since each of these tests of simple effects uses three degrees of freedom, we will follow up using pairwise comparisons.

### Ethical aspects

This study was approved by the Ethics Committee Center for Research and advanced Studies in Dentistry, and patients were informed about the research objectives through an informed consent form. All procedures performed in studies involving human participants were in accordance with ethical standards of the institutional research committee and with the Association Declaration of Helsinki 1964.

## Results

### Sociodemographic characteristics

The sociodemographic characteristics of the patients in the present study show that the average age was 13.7 ± 2.07 years. 64% of patients were women, and 36% were men. 18 % of the patients brushed once each day,  27 % brushed 2 times each day, and 55 % brushed 3 times daily (see Table 1).

**Table 1:** Characteristics of the patients included in the study by intervention group.

Characteristics	N = 35
**Sociodemographic**	
Age [mean (standard deviation)]	13.7 (2.07)
Sex	
Man	36%
Women	64%
Scholar	
High school second grade	25%
High school third grade	75%
Tertile of socioeconomic level	
I	35%
II	33%
III	33%
Salivary pH level [average] mean	
Basal	7.12
3 months	6.93
6 months	7.44
**Oral hygiene**	
Frequency use of toothbrush	
1 time a day	18%
2 time a day	27%
3 time a day	55%
Frequency of toothbrush change	
1 month	56%
3 months	31%
6 months	13%
Use of oral auxiliaries	27%
**Dietary habits**	
Sugar level	
Low (1 to 2 times a week)	11%
Medium (3 to 5 times a week)	60%
High (> 5 times a week)	29%
Level sugary drinks	
Low (1 to 2 times a week)	31%
Medium (3 to 5 times a week)	62%
High (> 5 times a week)	7%
Fast food and vegetables	
Low (1 to 2 times a week)	31%
Medium (3 to 5 times a week)	58%
High (> 5 times a week)	11%

### Multielemental analysis by ICP-OES

The results of the released concentrations of Ni and Ti in saliva and urine, as biological samples are observed in Table 2. The ICP-OES results for nickel in saliva, differences were observed (p < 0.05) for the comparisons: baseline vs. 6 months and 3 months vs. 6 months. For nickel in urine, differences were observed (p < 0.05) for the comparison: 3 months vs. 6 months. No significant differences were observed for the presence of titanium in saliva (p > 0.05). For titanium in urine, we observed statistically significant differences (p < 0.05) between all group comparisons (baseline vs. 3 months, baseline vs. 6 months, and 3 months vs. 6 months), statistically significant differences were found.

**Table 2:** Multiple ANOVA by groups between the average concentrations of nickel and titanium in saliva, urine and pH salivary.

Mg L^−1^		
Groups	Difference	p value
**pH salivary**^**1**^		
3 months-basal	− 0.1818	0.000
6 months-basal	0.3212	0.000
6 months-3 months	0.5030	0.000
**Nickel in saliva**^**2**^		
3 months-basal	0.0020	0.458
6 months-basal	− 0.0061	0.031
6 months-3 months	− 0.0082	0.003
**Nickel in urine**^**3**^		
3 months-basal	− 0.0007	0.725
6 months-basal	0.0038	0.092
6 months-3 months	0.0046	0.013
**Titanium in saliva**^**4**^	WSumCRank	Skillings Mack (Friedman test) no significant
3 months-basal	− 10.66	
6 months-basal	8.06	
6 months-3 months	2.60	
**Titanium in urine**^**5**^		
3 months-basal	0.0020	0.002
6 months-basal	0.0033	0.000
6 months-3 months	0.0013	0.014

Delta-method Pairwise comparisons of adjusted predictions.

^1^ANOVA p = 0.0000, Huynh–Feldt epsilon p = 0.0000, Greenhouse–Geisser epsilon p = 0.0000, Box's conservative epsilon p = 0.0000.

^2^ANOVA p = 0.0091, Huynh–Feldt epsilon p = 0.0091, Greenhouse–Geisser epsilon p = 0.0104, Box's conservative epsilon p = 0.0319.

^3^ANOVA p = 0.0342, Huynh–Feldt epsilon p = 0.0415, Greenhouse–Geisser epsilon p = 0.0439, Box's conservative epsilon p = 0.0689.

^4^Skillings Mack (Friedman test) p = 0.3047.

^5^ANOVA p = 0.0000, Huynh–Feldt epsilon p = 0.0006, Greenhouse–Geisser epsilon p = 0.0006, Box's conservative epsilon p = 0.0009.

### Determination of salivary pH

The values were obtained for salivary pH, in the multiple comparison analysis for pH, we observed statistically significant differences (p < 0.05). between all the group comparisons (basal vs. 3 months, basal vs. 6 months and 3 vs. 6 months) (see Table 2).

**Figure 1:**
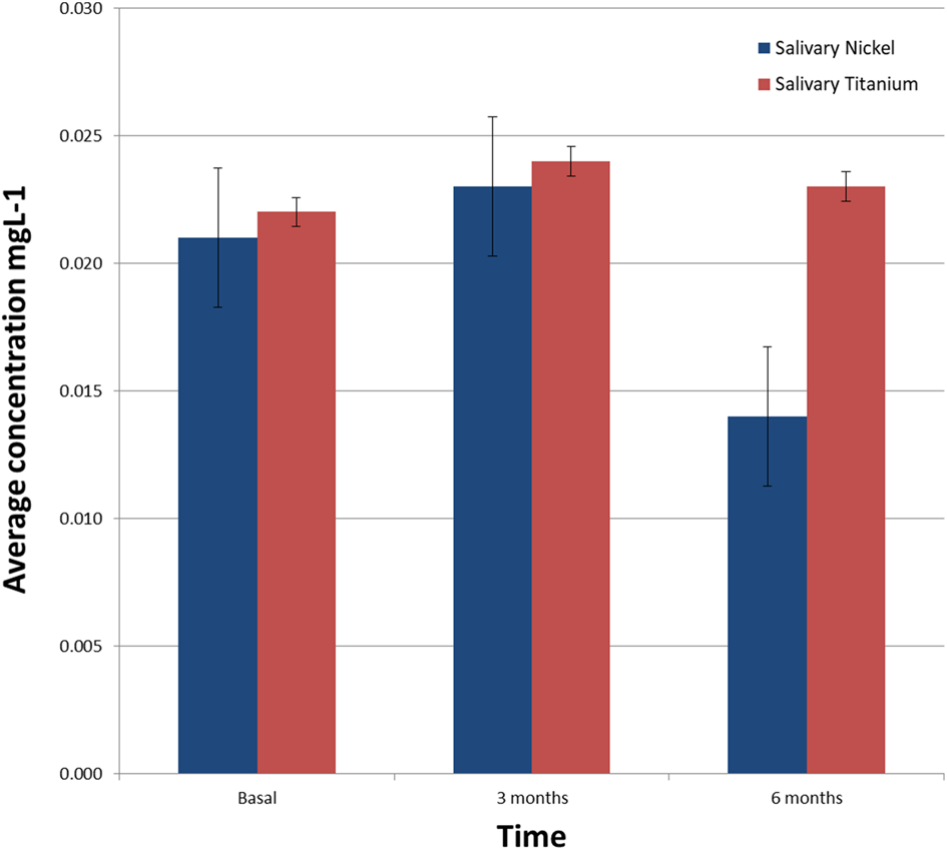
Concentration of Ni and Ti in saliva in each stage of intervention. The error bar represents the ± standard deviation (SD).

**Figure 2:**
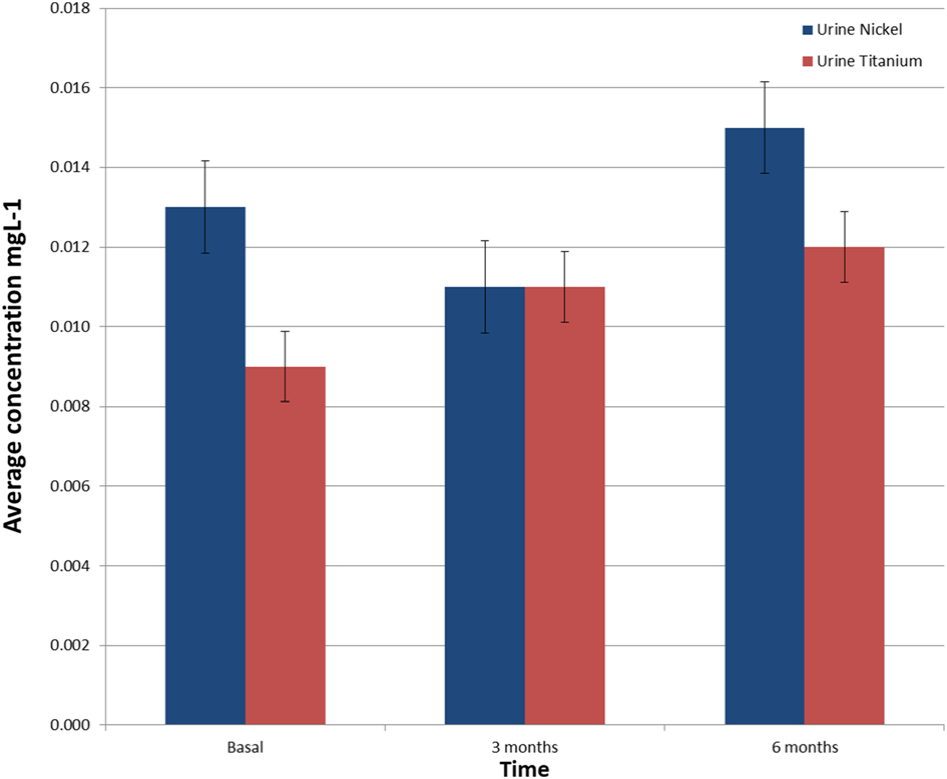
Concentration of Ni and Ti in urine by intervention stage. The error bar represents the ± standard deviation (SD).

The Ni concentrations released in each intervention stage are shown in Fig. 1. The Ni concentration in the basal stage (prior to the placement of the fixed appliances) sample levels of 0.021 ± 0.011 mg L^−1^. After three months, this release increased to 0.023 ± mg L^−1^, while at 6 months a significant decrease is observed, reaching 0.014 ± 0.008 mg L^−1^. For the Ti release in saliva for each stage of intervention, the baseline measurement was 0.022 ± 0.009 mg L^−1^, at 3 months, it increased to 0.024 ± 0.012 mg L^−1^, and at 6 months, it was 0.023 ± 0.010 mg L^−1^. The ions released concentrations in urine at each stage of the intervention is present, all the results for average concentrations are represented as mean ± standard deviation (Fig. 2).

The nickel concentration in the basal stage (prior to the placement of fixed appliances) has levels of 0.013 ± 0.009 mg L^−1^. After 3 months, this release decreases to 0.10 ± 0.006 mg L^−1^, while at 6 months, a significant decrease is observed, reaching 0.015 ± 0.006 mg L^−1^. For the Ti release in urine for each stage of intervention: at baseline, it was 0.009 ± 0.003 mg L^−1^, at 3 months, it was 0.011 ± 0.002 mg L^−1^, and at 6 months it was 0.012 ± 0.002 mg L^−1^, all the results for average concentrations are represented as mean ± standard deviation.

### Analysis by SEM and EDS

**Figure 3:**
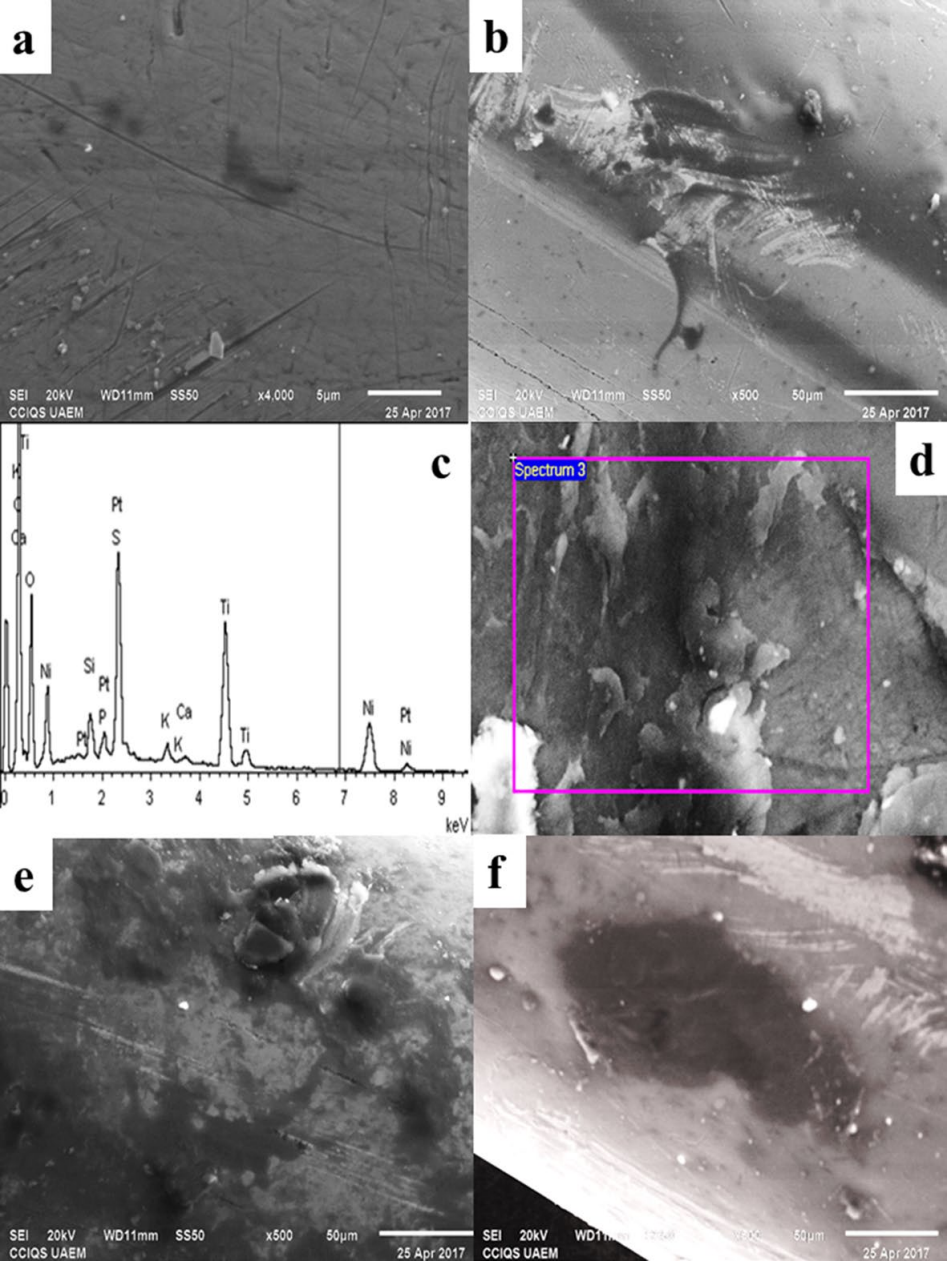
The 0.016″ Ni–Ti arch analyzed using SEM. (**A**) Reference sample surface arc, increase ×4000. (**B**) Surface RCO after 1 month cabbage or cation in oral cavity. (**C**) EDS of the arch after 1 month of placement. (**D**) Arc surface after 1 month of placement, at higher magnification. (**E**) The arc surface is observed with the presence of corrosion zones. (**F**) Corrosion surface, increase of ×500.

The 0.016″ Ni–Ti arch of the reference sample before placement in the oral cavity is observed in Fig. 3A. It shows a smooth surface without signs of corrosion. The micrograph Fig. 3B shows the corrosion delimited is observed in the micrograph of the 0.016″ Ni–Ti arch compared to the corrosion free micrograph. The EDS spectrum of the same arch is shown in Fig. 3C the contact between the friction arch and the oral environment presented the following concentrations C (37.84%), O (17%), P (0.73%) and K (0.82%) compared to the arch sample basal. Ti (20.38%) and Ni (21.70%) were detected with increasing time. The weight loss of Ni and Ti corresponds to the corrosion on the surface of the arch. Figure 3D show the morphological characteristics of 0.016″ Ni–Ti that was placed for 1 month in the oral cavity. There are fragments of flat particles, and presence of corrosion. The Fig. 3E,F show an increase of 500 and 600×, and an irregularly shaped structure is observed with the presence of corrosion zones with a size of approximately 50 μm.

**Figure 4:**
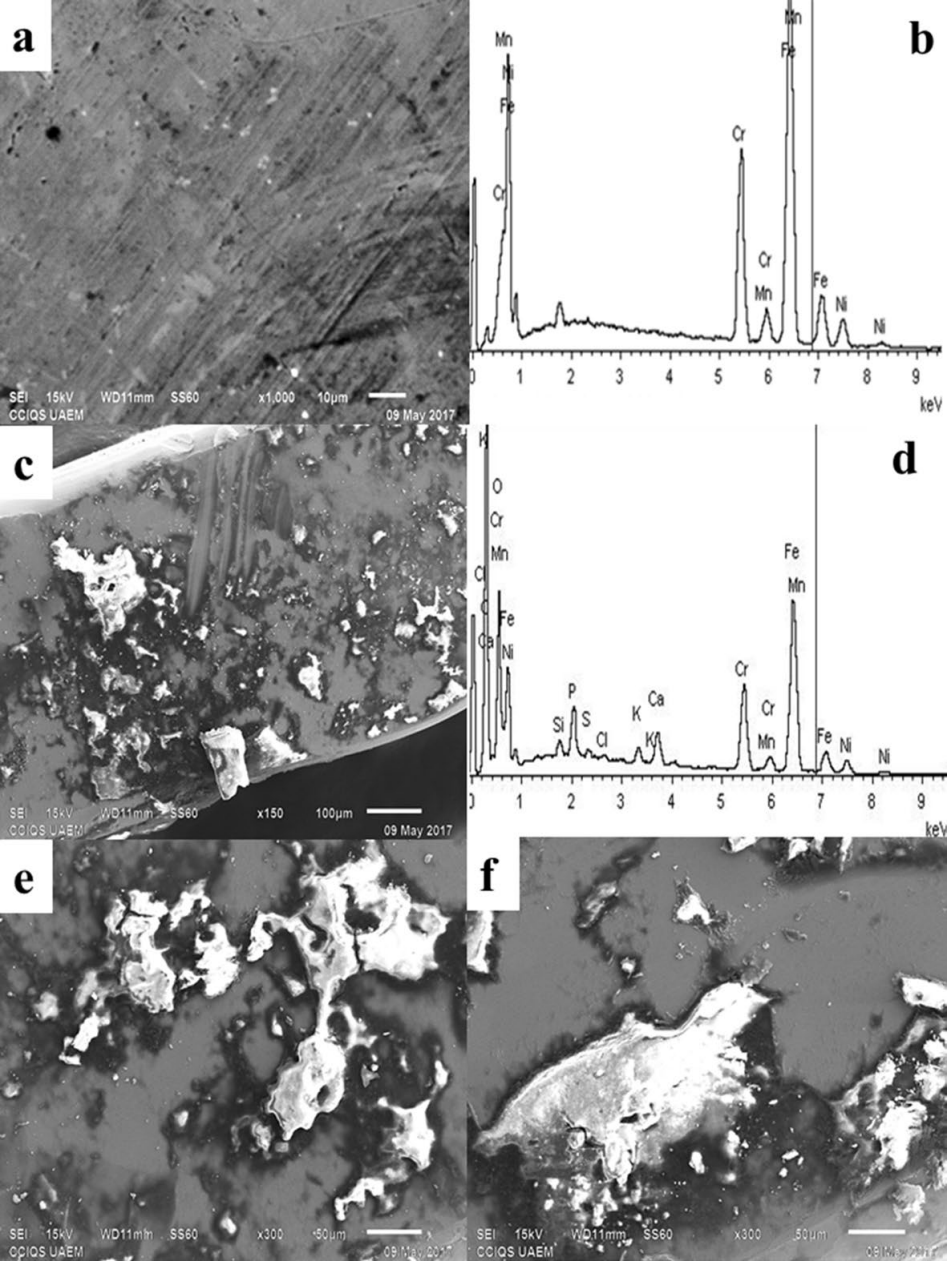
The 0.016 × 0.022″ stainless steel arc using SEM. (**A**) Reference sample of the arc surface. (**B**) EDS of the reference sample. (**C**) Arc surface after 1 month placement in the oral cavity. (**D**) EDS arc stainless steel after a month placement. (**E**) Surface arc increase ×300 and size of 50 µm. (**F**) Surface corrosion with increased ×300 and a size of 50 μm.

Figures 4A,B correspond to the micrograph and spectrum of the reference sample of the stainless steel arc with caliper (0.016 × 0.022″) before its placement in the buccal medium. The EDS indicates the weight percentages corresponding to each one of the elements present in the sample: Mn (1.39%), Fe (69.95%) and Ni (8.77%). Figure 4C show the corrosion. Figure 4D shown values EDS of Ni (3.81%) and Fe (29.40%) decreased compared to the reference wire. Figure 4E,F at 300× show areas where corrosion occurred in the arches of the wire.

## Discussion

In the present study, the release of metal ions was evaluated using the ICP-OES technique to measure the concentration of potentially biodistributed traces in the body. This method was able to compare the value with a permitted toxic limit. It was determined that metal ions are released only in the initial stages of treatment, and this is supported with the literature^30^. The main factors that impact are the time and bioelimination path which should be taken into account with the use of fixed appliances. These aspects are important for understanding allergic reactions that can occur in the body. According to the World Health Organization, the daily doses allowed for each element analyzed in this study are: Ni 25–35 μg day^−1^, Cr 50–200 μg day^−1^ and Ti 25–30 μg day^−1^^31^. Ni is absorbed in the gastrointestinal tract, and the main route for disposal is the kidneys, where nearly 90% is excreted in urine. The biological material is also removed by saliva and sweat, which could contribute to increased excretion at higher temperatures^32^. Barrett et al*.* studied the relationship of the release of Ni in saliva with the use of Ni–Ti and stainless steel appliances; they demonstrated that there is an increased Ni concentration during the first weeks after the placement of orthodontic appliances that subsequently decreases. On the other hand, the Cr release increased during the first 2 weeks and stabilized in the following 2 weeks^33^. Amini et al*.* evaluated the Ni and Cr concentration in saliva during the stages of orthodontic treatment and noted that there is a greater release of salivary Ni, while Cr values did not show significant differences^34^ The results of this investigation are correlated with the previous ones, where the release of Ni and Ti in saliva increases significantly after 3 months of orthodontic appliance placement in relation to the control group.

In this investigation, the patients used Ni–Ti arches at the beginning of the treatment (leveling), the duration of this stage is approximately 3 months, therefore Ti was evaluated up to 6 months because it can be released from other orthodontic attachments present in the mouth, and not only from the stainless steel arches at this stage. In addition, the saliva Ni concentrations obtained after 6 months indicated a statistically significant increase. In the case of Ti, did not statistically significant difference was found at 6 months. Although the concentration obtained from the metal ions released does not present toxicological risks, the release increases after the placement of appliances and gradually decreases after 6 months. Freitas et al*.* evaluated the release of Ag, Cd, Cu, and Zn in saliva at different times (10 min, 24 h, 7 days, 30 days, and 60 days) after the placement of fixed appliances. These elements reached concentrations that imply potential toxicity risk, with Cu concentrations (70.60 μg L^−1^) and Zn (0.07 μg L^−1^). The concentrations obtained in similar stages of time for the release of Ni in saliva differ from those reported by Freitas et al*.* did not presented toxic levels in any of the elements studied^35^, however Maspero et al*.* showed the chemical properties of titanium TSME applied to patients allergic to nickel has been an alternative in individuals with a history of allergic reactions to metals^36^. Ousehal et al*.* assessed concentrations of Ni and Ti saliva before and after the placement of the Ni–Ti arc. They did not found significant differences after 8 weeks. However, in this investigation, the values of Ti in saliva presented a significant difference at 3 and 6 months^37^. Chojnacka and Mikulewicz measured the release of Ni, Cr, Co, Fe, and Ti in urine, blood, and saliva at different intervals from day 1 to 2 months. They observed an increase in the concentration of Ni and Cr in saliva after the insertion of orthodontic appliances^38^.

Yassaei et al*.* found that Ni and Cr concentrations increase in saliva in the first 2 months of treatment orthodontic. These results coincide with other studies indicating that the Ni concentration in saliva was increased after placing fixed appliances, and exposure of saliva generates Ni bioaccumulation in the body^39^. However, Kararia et al*.,* did not found a significant concentration of Ni and Cr in saliva^40^. In the case of the bioaccumulation of ions in urine, Menezes et al*.* indicated that the concentration of Ni increased after 2 months of the placement of orthodontic appliances^30^. Fraunhofer and Giokaa et al*.* have reported that brackets of nominally the same composition can exhibit significantly different corrosion behavior in an in vitro artificial saliva exposure test^41,42^.

Yanisarapan et al*.* reported a limitation of the saliva for in vitro studies, because it plays an important role in reducing the concentration of metal ions released from orthodontic appliances^43^ and it presents a corrosive environment and cell and tissue reactions in the oral cavity, however the results obtained in our study make it more significant because Ni and Ti concentrations reached the highest levels at 3 months.

Bo Chen et al*.* showed that after 1 month of treatment, the urinary Ni concentration increases^44^. In the case of urinary Cr, this increases occurred up to 6 months Amini et al*.* observed that Ni concentrations in urine rise significantly after placing stainless steel arches for orthodontic treatment^32^. In terms of salivary pH, the results of this investigation indicate a decrease at 3 months after treatment. However, after 6 months, the pH increases again, remaining at an alkaline pH.

Lara-Carrillo et al*.* reported that the orthodontic appliances increase the retention of the plaque, causing high levels of concentration of hydrogen ions in the oral environment, and this modifies the pH. However, this external phenomenon stimulates salivary secretion and increases the concentration of bicarbonate ions and the modified pH. Saliva works as a shock absorber of physiological response to maintain oral health in adverse situations. Therefore, the buccal medium manages to adapt to the presence of a foreign body, increasing the salivary flow and modifying the salivary composition to raise the pH and buffer capacity^45^.

Regarding the evaluation of surface characteristics and structural changes for the 0.016″ Ni–Ti arch with orthodontic treated patients, these results show that there is corrosion on the surfaces of the arches in treatments of prolonged periods. The corrosive effect involves the loss of Ti and Ni. In addition, in the micrographs of the arches, striated structures are observed that indicate this corrosion. For years, 0.016 × 0.022″ stainless steel arches, the EDS analysis shows concentrations less than Cr compared to the control arc.

Močnik et al*.* evaluated the properties of Ni–Ti arcs in the presence of artificial saliva and concluded that the corrosion susceptibility increases when the pH is acidic. They also observed ion release at a controlled temperature of 37 °C to simulate the temperature of the oral cavity. In this case, they found that the Ni ions were below of the 0.5 limit μg week^46^.

On the other hand, Wang et al*.* demonstrated that Ni–Ti arcs stuck in different salivary pH environments at a constant temperature of 25 °C, showing corrosion by analyzing micrographs obtained by SEM^47^. In addition, Yamaguchi et al*.* confirmed that the saliva at room temperature and with an acid pH generated an environment that was more sensitive to corrosion in the arches (Ni–Ti)^48^. Therefore, it is concluded that salivary pH in the decomposition of the protective layers and degradation and mechanical properties. On the other hand, Lee et al*.* added fluoride ions in artificial saliva, simulating the effect of toothpastes containing fluoride, they demonstrated that the ions proved detrimental to the corrosion resistance of orthodontic arches containing Ti in different copper. Alloy ions (Ni–Ti, Ni-Ti-Cu, Ti-Mo-Zr-Sn-Nb and Ti), especially in the highest concentration of fluoride (0.5% NaHF)^49^.

In the present study, the salivary pH of patients was determined and correlated with the release of Ni and Ti. The results show that at 3 months, the pH is modified, causing an oral buccal medium that is mainly influenced by the hygienic-dietary changes and the dento-bacterial plaque accumulated by the increase of retentive surfaces of fixed orthodontic appliances. In a second evaluation, at 6 months, the pH is normalized to an alkaline medium. However, these studies utilized artificial saliva, which is unlike our study that collected data from patients. Stimulated saliva can be affected by many physiological variables, such as diet, oral hygiene, health conditions, and salivary flow rate, as observed in the results.

One limitation of this study was the short evaluation period. More time intervals are needed in order to meet the bioabsorption and bioelimination of metal ions during the complete duration of orthodontic treatment. This could be helpful to asses if there is a concentration that is bio accumulated in the body by the same mechanism.

## Conclusions

Orthodontic appliances release a measurable amount of Ni and Ti when they are placed in the oral cavity. According to the results of this study, it can be concluded that although there is an increase in the release of ions (Ni and Ti) in saliva 3 months after placement, when it reaches its peak release, the concentrations are within acceptable limits. Therefore, the values of Ni and Ti concentrations in the urine increased at 3 and 6 months after treatment.

The salivary pH turns acidic 3 months after the placement of the abutments. However, after 6 months, it returns to alkaline conditions.

The SEM analysis showed that both stainless steel and Ni–Ti arches have the same susceptibility to metal corrosion.

These results are sufficient to prompt further research on bioconcentration and bioaccumulation of Ni and Ti in the oral cavity. Systemic toxicity may be significant although Ni concentrations are permitted, and its presence is an existent threat to the integrity of oral tissues and the use of these metals in clinical practice.

## Data Availability

The data sets generated during and analyzed during the current study are available from the corresponding author on reasonable request.
